# Supplementary material for: CyberKnife in Pediatric Oncology: A Narrative Review of Treatment Approaches and Outcomes
Source: Curr Oncol. 2025 Jan 29;32(2):76. doi: 10.3390/curroncol32020076 (PMC11854067; doi:10.3390/curroncol32020076)
Supplement: Supplementary file 1 [file curroncol-32-00076-s001.zip › curroncol-3379015-supplementary.pdf]

**Supplementary Materials:**

**Table S1: Checklist Items.**

| Section/topic | # | Checklist item | Reported on page or line # |
|---|---|---|---|
| **TITLE** | | | |
| Title | 1 | Identify the report as a Narrative Review | Page 1 |
| **ABSTRACT** | | | |
| Unstructured summary | 2 | Provide an unstructured summary including, as applicable: background. Objective, brief summary of narrative review and implications for future research, and clinical practice or policy development. | Page 1 |
| **INTRODUCTION** | | | |
| Rationale/background | 3 | Describe the rationale for the review in the context of what is  already known | Page 2-4 |
| Objectives | 4 | Specify the key question(s) for the review topic | Page 2-4 |
| **METHODS** | | | |
| Research selection | 5 | Specify the process for identifying the literature search (e.g. years considered, language, publication status, study design, and databases of coverage | Page 4-9 |
| **DISCUSSION/SUMMARY** | | | |
| Narrative | 6 | Discuss: 1) research reviewed including fundamental or key findings, 2) limitations and/or quality of research reviewed, and 3) need for future research. | Page 9-13 |
| Summary | 7 | Provide and overall interpretation of the narrative review in the context of clinical practice for health professionals, policy development and implementation, or future research. | Page 13 |
